# Lecture upon the First Three Paragraphs of the *Organon*

**Published:** 1891-04

**Authors:** Walter S. Hatfield

**Affiliations:** Cincinnati, Ohio


### LECTURE UPON THE FIRST THREE PARAGRAPHS OF THE *ORGANON.*

WALTER S. HATFIELD, M. D., CINCINNATI, OHIO.

GENTLEMEN:—It would be difficult to find fewer words containing more truth than those found in paragraph first of the *Organon:*

"The physician's highest and *only* calling is to make sick people well, which is called healing."

If physicians would only give heed to the truth contained in that single paragraph and cease trying to perform impossibilities, they would be benefited thereby. There were people in Hahnemann's lifetime, the same as now, who always searched for the impossible. Not that scientific research should be abandoned, but it should be pursued in the interest of human kind.

The healing of the sick should be our motto, and when we go beyond that we are getting out of our sphere.

There must necessarily be experiment in medicine. And the difference between the new and old school is the former experiment upon the healthy people and the latter upon the sick.

It is more safe to experiment upon the healthy, because there is no danger of injury—that is, if the toxic effects are not in view—while in the sick valuable time might be lost, besides the experiments would not be satisfactory.

The extent of the investigations of the old school is to get as near to the poisonous effects as possible without producing death, because, with them, the largest possible dose is the best. With the exception of the foremost men in that school, some of them, in treatment, reduce the dose to nearly homœopathic dimensions, some of them even using homœopathic preparations. But the rank and file of that school are still of the opinion, "If a small dose will do good, a larger one will do more good."

In the treatment of the sick, drugs are generally compounded, and that without reason. If a patient dies, which often happens, they are not aware which of the several ingredients caused death. Or, if recovery takes place, which is sometimes the case, they are at a loss to know to what source they should attribute their success. All is as dark as before, so far as gaining any positive knowledge from the treatment is concerned.

They do not know how the medicines acted: which, if any, was antidoted by the other, or in any way interfered with in its action.

They have observed that some few drugs are incompatible; others claim that trouble can be overcome by certain methods of preparation.

But we know there is no way of preventing one medicine from interfering with the action of another within the system, when both are administered at the same time, or if the second be given before the action of the first is entirely spent.

We will see, as we advance, that nature is not in the habit of allowing two or more separate disease forces (either natural or drug disease) to attack the system at the same time. The weaker must give way to the stronger.

It is not the aim of the physician—the true healer—to experiment when called into the sick-room. He should have done with experiment and be prepared to administer to the sick that which will bring about a return to health. That is his *only* calling. We are also instructed, further along in the *Organon,* when and how to experiment, and when we have learned that part we will find the sick-room to be unknown to experiment.

Hahnemann wrote the *Organon* after years of experience, and no one could express in fewer words more truth than that which is found in paragraph first. He emphasizes the word "*only*" and makes it doubly forcible because, the *only* calling of the physician is to make sick people well. This constitutes the physician's life work.

Many physicians strive for notoriety, fame. The physician is fortunate in his freedom of action. In the treatment of the sick he is at liberty to make use of any means whatever, and if he has gained notoriety he has attained his aim. If the patient recovers, well and good; but if he does not it is of little difference. It is sufficient to know that the physician did all that could be done, no matter how absurd it may have been.

We have only to mention cases to verify this. Our own ex-President Grant, also Emperor Frederick, of Germany. These cases are still fresh in our minds. According to the published reports, the cases were similar, and the treatment about the same (highly scientific ?) and the results identical. But the attending physicians became famous in spite of the unfavorable terminations.

But if the distinguished patients had been treated according to the homœopathic law of similars and the result had been success, there would have been nothing thought about it, only that it would have proven (according to the popular belief) the illness to have been non-malignant. In fact, there was nothing the matter with the throat of either of them.

I speak from experience. During the past year I have been treating a patient who has been suffering on account of an unnatural growth upon the forehead, which was, in my opinion, a cancer. A professor of this College also saw it and, if I mistake not, was of the same opinion. Likewise everybody who saw it, so far as I know, thought the same. And the general friendly advice (such advice you will find always to be forthcoming from relatives, friends, and especially neighbors of the patient): If I were in your place I would have this or that done, or, my doctor, or doctor so and so, always does thus and so! and he is an older doctor than this one and he ought to know more about such things. Or, my mother, grandmother, or aunt has raised a large family and always did this or that; this medicine isn't strong enough for the child; or, it may do for children, but it is too weak for grown people, and especially in such a severe case as this you want something that will take right hold and make you feel as though something was being done. And the general conclusion of the whole matter is, you had better send for Dr. ——; he is our doctor, and I know he is a good one. Such advice you will find always proffered the patient and family. But the general advice in this case was to have the growth removed. All were positive on that point. It should be removed by excision while there was yet time, and if allowed to remain the eye would be lost, and, in fact, life itself would soon be made miserable, and a slow and painful death was all that could possibly be expected.

But, strange to relate, the growth has been almost wholly removed by homœopathic remedies, and now the general opinion is that it never was a cancer. And I believe Homœopathy will succeed in its entire removal, and with the removal of that product the whole disease will probably be extinguished—driven from the system.

On the other hand, had the knife been used, the tumor or unnatural growth would have been successfully disposed of, no doubt. But what of the future? The disease would have remained within the system the same as before, and it might have been excited to greater energy.

It is not sufficient that we should succeed in relieving pain by means of the usual nerve depressants, for the pain is often one of the most important symptoms to guide us in the selection of the remedy. But the properly-selected remedy will generally relieve the pain, and with the disappearance of the pain often the system is relieved of all trace of disease.

And often pain is relieved almost instantly.

You will be astonished at the quick response from the well-selected remedy.

If, in your anxiety to relieve pain, you should administer Morphia you will find yourselves in the dark. The symptoms will be clouded and the after-treatment will be unsatisfactory.

But take the symptoms for your guide and you will be successful. Relieve the pain by means of the indicated remedy and the disease will be cured. I will give you the history of a case.

Two years ago, a man, about fifty years of age, night-watchman on the river, was obliged to be out during a heavy rain, and the weather turning suddenly cold he contracted a heavy "cold." The attending physician said it was pleurisy, and the man was treated accordingly. After several weeks of treatment the doctor failed to relieve him, and of his own accord ceased his attendance.

It was not for want of money, the doctor had been his family physician for years and had always been paid. But it was because he could do him no good.

The doctor was all at sea. He could make no diagnosis. He had relieved the pleurisy, the symptoms had changed, and he was off the track. You are aware that when an old-school physician cannot name the disease he is in a dilemma. Being unable to make a diagnosis, the doctor was equally unable to prescribe satisfactorily. And after the several weeks had gone by, the patient seemed worse.

And the doctor failing to cure, another old-school doctor was sent for, and his conclusion was, the patient was suffering on account of malaria and general debility, with neuralgic complications, and prescribed accordingly, and assured the man he would be all right again very soon.

Two weeks later I found the following condition and symptoms.

A 200-pound man reduced to about 140 pounds. Unable to eat anything nourishing. Bowels constipated; a movement only once in several days. Lightning-like pains down the spine, around sides, in abdomen, and down lower extremities. These pains would generally come in paroxysms, in the evening, later in the night, and toward morning. Sometimes they were almost continuous and so severe as to cause loud outcry. And with the pain vomiting was generally present, and of course sleep was impossible.

During the paroxysms he would oblige his attendants to take him out of bed, sit him in a chair or hold him up while he endeavored to walk about, but in his weakened condition it was almost impossible.

Nux-vomica was my first prescription on account of previous drugging. The next day, after studying the case as well as I could, Magnes-phos. was given; improvement followed, but not marked, and in a few days, as the symptoms had changed somewhat, Aluminum metallicum was the next remedy. After its administration there was a marked change for the better, but on account of the nature of the disease the improvement was slow.

During the fifth week he could be up most of the time, sitting in the big chair, but it was several weeks before he could walk as well as before. His lower limbs were partially paralyzed for a time after.

In a few months, however, he was able to return to his work and has seemed well ever since.

I concluded the patient was suffering from progressive locomotor ataxia. The diagnosis did not help me in the treatment, only in a general way helped me to find the remedy, for the totality of the symptoms guided me.

It might be a question why the Nux-vom. was given.

Whenever a case comes from old-school hands it is a good rule to give Nux-vom. generally, to antidote the drug action, and often it will help to clear up the case if the symptoms are not well defined. But it is not always best. Perhaps if the Aluminum-met. had been given in the first place the improvement might have been greater from the first, but I did not see the remedy so clearly indicated until the constipation was so marked. Then I was led to prescribe that remedy with the best of results.

Had I given the patient Morphia for his pain and cathartics for his bowels, etc., I doubt not that to-day he would be a burden to himself and family.

Let me tell you of another case received from old-school hands that did not receive Nux, for, as I said before, it is not always necessary to use it. It was a case of diphtheria. The patient, a boy eight years of age, was summering at Epworth Heights camp-ground. During an evening entertainment he fell asleep, rolled off the seat, and lay on the ground an hour or so before he was discovered. Nothing was thought of it until, two days later, he was taken with a high fever, neck a little stiff, thirsty, throat sore, etc. An eminent physician on the grounds said it was a bad cold and he would be well in a day or two, and prescribed for a cold. This was Saturday. On Sunday the doctor saw him again, prescribed for a cold as before, with sore throat. Next morning (Monday), on examining the child, the doctor told the parents they had better take him away, and also told them their family doctor would probably tell them it was diphtheria.

He therefore wrote the family doctor a note stating what he had given: "Potas-chlor., Tr. Acon., Tr. Iron, and Quinine freely." But instead of taking him to the family doctor they brought him to me. Since then *I* have been the family doctor. The present condition is this: Neck somewhat stiff, on account of the swelling of the throat and jaws, throat well filled with a white looking membrane, more on left side, posterior nares filled with the membrane, as far as could be seen; a clear watery discharge from nostrils, not very thirsty, pain in throat, began on *right side*, tending toward the left.

The boy did not get Nux-vom., but instead Lycopodium$^{cm}$.

The next morning (Tuesday), much the same. Slept very well, except difficult breathing, on account of obstruction of the nose, much clear watery discharge from nose. ℞ Lycopodium continued.

Wednesday.—More pain in throat on swallowing spittle, less on swallowing drink (no solid food allowed). Pain more on left side; discharges from nose, foul odor from mouth, membrane less in throat. ℞ Lachesis$^{cm}$.

Thursday.—Membrane forming again in throat, nose completely obstructed with an exceedingly acrid watery discharge from nostrils, nose, and lips, sore on account of the acrid discharge. ℞ Arum-try.$^{20m}$.

Friday.—Membrane disappearing from throat, nasal passages clearer, nasal discharge less, no pain in throat. No change in prescription.

Saturday.—Much improved in every respect.

Monday.—Throat entirely clear. Nasal passages unobstructed, feels well in every way, nothing wrong, except soreness of nose and lips, which will soon disappear. Discharged.

In the treatment of diphtheria usually one remedy is sufficient to complete the cure, but not always.

It was plain, afterward, that the giving of Lachesis on Wednesday was a mistake. But Lycopodium had ceased to be of benefit, and the left side seemed so painful, together with the foul breath, led me to prescribe that remedy, whereas, a few hours of waiting would have shown the proper remedy to be Arum-try.

The whole case was more or less influenced by the previous medication.

The second paragraph of Hahnemann's *Organon* reads: "The highest ideal of healing is the speedy, gentle, and durable restoration of health, or the cancellation and annihilation of the disease in its whole compass; in the shortest, most reliable, and least-damaging way, according to clearly intelligible reasons."

It should be our aim and only thought in the treatment of the sick, to restore them to health, and to accomplish that end in the quickest manner possible. No harsh measure should be made use of, and when health is restored it may prove to be a permanent restoration.

A young girl came to me a few months ago, suffering on account of menstrual irregularities. In fact, she had not been well since having had diphtheria about eighteen months before. Before that she had never known any trouble. The method of treatment, pursued during the attack, was that of the ordinary gargles, swabbing, simulating an antiseptic character. The membrane was dislodged, and she succeeded in remaining here while two other children died of the same disease. But was she cured?

I do not believe she was. Had she been cured she would have had no trouble afterward. Many sad cases result from the mal-treatment of diphtheria. Long-lasting throat affections, paralysis, and impaired constitutions.

Malaria is another of those troublesome complaints, when there is suppression instead of cure, the result of treatment.

The amount of Quinine given for malaria alone is sometimes remarkable. One of my patients tells me a number of years ago he had malaria for a long time. His physician concluded he could cure him, if he (the patient) could stand the treatment. In desperation the patient promised to do or take anything the doctor might suggest or prescribe. As a last resort the doctor *prescribed fourteen hundred grains of Quinine*, to be taken *one hundred grains per day*, until all were taken. The task was begun but never finished.

He says he took eight hundred (800) grains, and the evening of the eighth day they took him home to die. It is his opinion that he had been very close to death.

He finally recovered from the overdosing of Quinine, and later on was relieved of the malaria, but has never been thoroughly well since. Every spring the malaria returns and he has at least one chill, and after that he can check it again, with some remedy given him by some old Indian or other ancient. The age is what gives it value, no doubt. But so far as good health is concerned, he will never enjoy that blessing again. I wish to impress upon you the great difference between suppression and cure of disease.

At the Cincinnati Hospital you have the opportunity to witness heroic treatment, and from what I can learn it *is* heroic.

I remember one old man at the Pennsylvania Hospital, who was given ninety (90) grains of Chloral per day for sleeplessness, and even then the result was not satisfactory.

How about the innumerable cases of cauterized chancres, chancroids, condylomatous growths, etc., injected urethræ causing strictures, and the numerous evils following the suppression of such vile disorders?

Where is the gentleness of such treatment?

Where is the genuineness of such cures?

Instead of their being *cured* they are only relieved; sometimes not even that. Many cases can never be cured afterward, because the system is too thoroughly undermined by the superficial suppressive treatment.

Hahnemann saw the evil attending the excessive use of drugs. And to that source he attributes a good portion of the world's misery. Homœopathy has caused the old school to prepare the dose so it can be taken without so much repugnance. It cannot always be overcome, yet there is some difference since Mark Twain's boyhood days.

While the drugs may be made more palatable now than then, they are generally just as severe in their action.

You will find some difficulty in giving medicine when you follow an old-school doctor in a case. The children are in the habit of having their noses held to compel them to swallow the stuff, and they conclude all medicines are alike.

Children have a horror of the doctor because they have learned to know that torture attends his coming. But when they have once learned the taste of the little sugar pills, you will find your stock of Sac-lac will not hold out. At least, that is my experience.

The good-will of the little folks you will always have, and that is a strong point in your favor with the rest of the family, if any of them are out with Homœopathy.

Paragraph third of the *Organon* says: "The physician should distinctly understand the following conditions: What is curable in diseases in general, and in each individual case in particular; that is, the recognition of disease (indicatio). He should clearly comprehend what is curative in drugs in general, and in each drug in particular; that is, he should possess a perfect knowledge of medicinal power. He should be governed by distinct reasons, in order to insure recovery, by adapting what is curative in medicines to what he has recognized as undoubtedly morbid in a patient; that is to say, he should adapt it so that the case is met by a remedy well matched with regard to its kind of action (selection of the remedy, indicatum), its necessary preparation and quantity (proper dose), and the proper time of its repetition. Finally, when the physician knows in each case the obstacles in the way of recovery, and how to remove them, he is prepared to act thoroughly and to the purpose, as a true master of the art of healing."

There is a great difference between an old-school doctor and a homœopathic healing artist. If a man is good at guessing he will do for the former. All that is necessary for the old-school physician is to make a reasonable guess at the name of the disease and follow that with another guess at treatment.

That is distinctly "regular."

There is no effort made at precision.

A dozen different old-school doctors are likely to write a dozen different prescriptions for the same case. That is demonstrated by what Dr. Chapman, of Watsonville, Cal., did.

He wrote out the symptoms of a supposed case and sent the same to each of ten old-school doctors and ten homœopaths, and the result was, the ten homœopaths sent, each of them, a prescription for Lycopodium; while eight of the ten old-school doctors sent each an entirely different prescription. The other two sent none at all. Demonstrating to a certainty that to be regular one must be different.

But, how is it with the homœopath? The opposite is the case! He must be able to recognize diseased conditions which are curable in general, and in every individual case in particular. Nothing but the most rigid individualizing will do in Homœopathy. To be sure, one may be less careful and still do very well in Homœopathy, because we often do the right thing when we the least expect it. But, in generalizing we lower the probability of accomplishing good results nearly to the plane of the old school, which is the best they can do in the absence of all therapeutic law.

If the physician is capable of recognizing that which is curable in disease, he should also be able to discern in drugs (both in general and individually) that which is curative, and how to apply the same in the cure of disease.

Each remedy has its own individual action. While several remedies may have a similar general action, no one remedy can take the place of another in the perfect cure of a certain individual case. In a given case, if a remedy be administered, *not* the nearest or most perfect similar, that remedy may influence the case for good, but it can hardly result in a perfect cure.

In regard to the preparation of our remedies, it is not so necessary, as formerly, to understand all about that. Our pharmacies are capable and reliable, and we can get honest medicines.

But in Hahnemann's day, and even later, it was impossible to obtain homœopathic preparations, and of course the physician was obliged to prepare his own.

But the manner of administering the remedies, the proper dose, and the time to repeat are the question of utmost importance.

Whatever the preparation may be, mother tincture, low or high trituration, or potency, too much must not be given, and it must not be repeated too often.

Some use the mother tinctures and lower triturations and potencies, and never go higher, while others use both low and high. And still others use almost exclusively the highest. The success of each one depends upon "how" the prescription is made. If it is made according to the law of similars, the result is always satisfactory, but if made upon general principles then disappointment often follows. And Homœopathy gets the blame. Some physicians repeat the dose too often and are disappointed also.

When we get further along, we will learn from the *Organon* that when once the system is sufficiently influenced by the remedy given, then medication should be stopped until we are certain the improvement has ceased.

And, furthermore, anything whatsoever that may hinder or prevent recovery should be able to be seen and the manner of removal should be known to the physician.

There is so much depending upon the physician in each and every case. It is not only the giving of the medicine, but innumerable things which should claim his attention. And he should be able to comprehend and guard against those hindrances to recovery.

Therefore, gentlemen, do not for a moment think that the life of a physician, your chosen life-work, is all sunshine, and there is nothing difficult to perform.

Unless one has his whole soul in the work, he may find it exceedingly irksome.

